# KIDS OUT! Protocol of a brief school-based intervention to promote physical activity and to reduce screen time in a sub-cohort of Finnish eighth graders

**DOI:** 10.1186/s12889-015-2007-8

**Published:** 2015-07-10

**Authors:** Anne-Mari Jussila, Tommi Vasankari, Olavi Paronen, Harri Sievänen, Kari Tokola, Henri Vähä-Ypyä, Anna Broberg, Minna Aittasalo

**Affiliations:** UKK Institute for the Health Promotion Research, Kaupinpuistonkatu 1, Tampere, 33500 Finland; School of Health Sciences, University of Tampere, Tampere, Finland; Aalto University, Espoo, Finland

**Keywords:** Intervention, Evaluation, Physical activity, Sedentary behavior, Adolescents, School, Health education, RE-AIM

## Abstract

**Background:**

Adolescents’ physical activity (PA) is decreasing and sedentary behavior (SB) increasing alarmingly. Insufficient PA and excessive SB are both related to various health risks indicating that interventions to promote adolescents’ PA and to reduce their SB are needed. Schools have a great potential to reach most adolescents, and in Finland health education (HE) as stand-alone subject provides an excellent platform for health promotion. This paper describes the protocol and evaluation (RE-AIM) of an intervention developed for three HE lessons to increase PA and reduce SB during leisure among 8^th^ graders.

**Methods/Design:**

All city-owned secondary schools in Tampere (n = 14) were invited to the study and were randomized in pairs to intervention (n = 7) and comparison group (n = 7). A specific content on PA and SB based on Health Action Process Approach model was integrated into routinely scheduled three HE lessons with the help of educational material: SoftGIS-questionnaire followed by feedback views on adolescents’ current PA and SB, FeetEnergy-homework leaflet for adolescents, FeetEnergy-video in YouTube, FeetEnergy-poster for classroom and FeetEnergy-leaflet for parents. In the comparison group standard HE lessons were held. The primary indicators of Effectiveness are changes in PA and SB and in their psychosocial factors as well as in parental interference with PA and SB. The measurement points are baseline, 4 weeks after the intervention and 7 months from baseline, the last indicating also the measurement point for individual level Maintenance. The measures are accelerometers, 7-day activity diaries and questionnaires. The evaluation of Reach, Adoption and Implementation is based on the data collected during the intervention. Maintenance at organizational level is assessed two years after the intervention with a questionnaire to the HE teachers. The intervention was implemented in 2012 and the last measurements to assess organizational Maintenance were conducted in the end of 2014. A detailed description of the protocol and evaluation is provided to enable replication and better understanding of the findings, which will be reported in 2015.

**Discussion:**

The findings will add our current knowledge about the feasibility and effectiveness of integrating simple structured elements into the HE lessons to increase PA and reduce SB in adolescents.

**Trial registration:**

NCT01633918 (June 27^th^, 2012)

## Background

Physical activity (PA) in youth is beneficial for musculoskeletal and cardiovascular health, obesity adiposity, blood pressure, metabolic syndrome, bone mineral density, anxiety, depression [1–4] and academic performance [4, 5]. To achieve these benefits children and youth should engage in moderate-to-vigorous-intensity PA at least one hour daily [2]. In Finland 17 % of 15 -year-old boys and 10 % of girls meet the current guidelines according to the HBSC-survey [6]. According to a more recent study with objective accelerometer data on 130 Finnish adolescents 17 % of the 13-14 year-olds meet the recommendation [7].

According to HBSC-survey 59 % of Finnish 13-year-old girls watched TV two hours or more per day on weekdays, 12 % played computer games and 55 % used the computer otherwise [6]. In 13-year-old boys the corresponding percentages were 60 %, 49 % and 44 %. During these popular leisure activities adolescents are usually sitting. On weekdays the amount of sitting further increases because of school. According to accelerometer-based data, 45 minutes of the 60-minute lesson is spent sedentary in Finnish secondary schools [7]. It is therefore not surprising that Finnish adolescents spent on average 10 hours in sitting on a weekday [7]. The findings are similar in other developed countries [8]. Excessive sedentary behavior (SB) has been found to be associated with unfavorable body composition, decreased fitness, lower self -esteem, social behavior and decreased academic performance [9, 10].

One reason for the increase of SB among adolescents can be the decrease of active commuting to school [11–13]. According to the Finnish National Travel Survey [14], young people have replaced walking and cycling with mopeds and microcars. Active school commuters in general tend to be more physically active than passive commuters [15, 16]. Cycling to school alone is related to better cardiovascular fitness in children and adolescents compared with their peers using motorized forms of transport [17].

Based on above facts, ways to promote adolescents’ PA and to reduce their SB are needed. School environment offers a good opportunity for promotion and reaches majority of the target group. In previous studies school-based interventions have had positive effects specifically on the duration of PA and television viewing [18]. Especially multi-component approaches including family involvement have shown promising results [19, 20]. In Finnish secondary schools health education (HE) as a stand-alone subject in addition to physical education provides exceptionally favorable platform for PA promotion. However, former studies utilizing HE lessons in PA promotion have not been reported in Finland.

This paper describes the protocol and RE-AIM evaluation [21] of a Kids Out! –intervention, which aims to increase leisure PA and reduce SB among 8^th^ graders by integrating multi-component approaches into three routinely scheduled HE lessons in secondary schools.

## Methods/Design

### Schools

In Finland, the compulsory schooling is public-funded and maintained by the municipalities. Secondary school includes grades 7 through 9 and the age of 8^th^ graders is 14-15 years. The intervention was conducted in the city of Tampere, which is located in the south-western part of Finland and comprises of more than 200,000 inhabitants. The Tampere City School Board gave general approval for the study in all city-owned secondary schools (n = 14). The private-owned (n = 3) and government-owned (n = 1) schools were not approached because they had different school administration. After the general approval the recruitment of individual schools included three stages: 1) a short oral presentation about the intervention in a principal meeting, 2) visits to the individual schools for more detailed information and, 3) a written agreement from the principals of the individual schools on participation. After the recruitment all city-owned 14 secondary schools agreed to take part in the intervention. The study plan was approved in the Ethics Committee of the Tampere Region, University of Tampere, Human Sciences (running number 6/2012).

### Randomization

The participating schools were arranged into pairs according to their number of students, location (urban/suburban) and proportion of students walking or cycling to school. The latter information was obtained from the data collected via web-based Tampere Physical Activity Survey, which had been conducted in the previous fall to all 8^th^ graders (n = 1638) in the city-owned secondary schools [22]. The schools in each pair were then randomized into either intervention (n = 7) or comparison group (n = 7). Individual-level randomization was not used to avoid contamination of the groups.

### Power calculations and sample size

Power calculations were also based on the Tampere Physical Activity Survey [22]. According to the power calculations 54-74 students from each secondary school was needed to discover the between-group difference of 0.5 days in the weekly number of days accumulating the maximum of two hours of screen time and in the weekly number of days accumulating the minimum of one hour of moderate-to-vigorous-intensity leisure PA with intra-cluster correlations of 0.005 and 0.01, standard deviation of change of 2.0, significance level of 0.05 and power of 80 %.

### Intervention

**Fig. 1:**
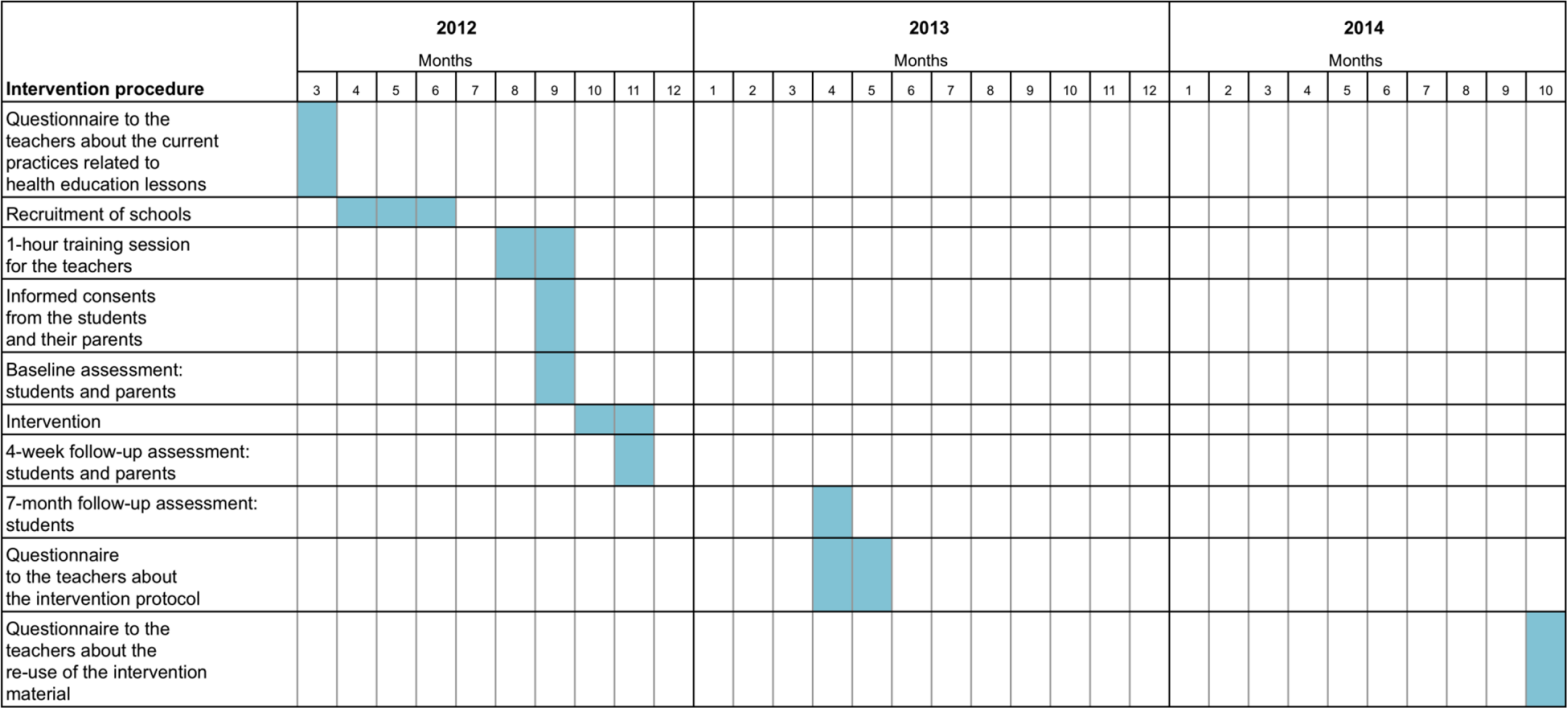
Timetable of the Kids Out! -intervention

The timetable of the intervention procedure is presented in Fig. 1.

#### Delivery

The intervention was planned to take place during three routinely scheduled HE lessons, which were preferably no more than one week apart from each other. According to the Finnish National Core Curriculum, the total number of weekly HE lessons per year is 38. This means one lesson per week and accumulates 114 lessons during the secondary school years. However, the schools are entitled to make their own curriculums at local level within the framework of national curriculum. One typical adaptation is that there is one weekly lesson per year at the 7^th^ and two weekly lessons per year at the 8^th^ grade. The current practices in the 8^th^ grades of Tampere were questioned from the HE teachers beforehand via e-mail and all variations of implementing HE lessons were acceptable for the intervention.

In the intervention group the HE lessons were integrated into the routine school practices and therefore all the 8^th^ grade students were exposed to them (n = 696). However, the students’ participation in the data collection was voluntary and an informed consent was required from them. The parents were informed about the study before and during the intervention in a written form and with the help of the teachers via electronic communication system between the schools and parents.

**Fig. 2:**
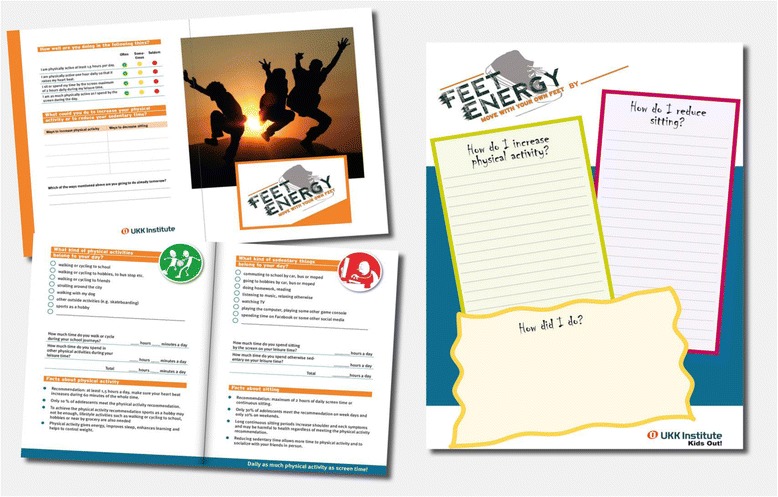
FeetEnergy -homework leaflet for adolescents and a classroom poster

The HE teachers, who are usually also responsible for physical education in the Finnish school system, delivered the intervention. Earlier studies indicate that it may be more effective to use physical education than general teachers as providers [18]. The teachers in the intervention group were trained for implementing the lessons. The training session took place at each individual school and lasted for one hour. During the session the teachers were introduced to FeetEnergy-material, which was specifically developed for each lesson (Fig. 2). The teachers received also a Teacher’s Manual, which provided them a detailed description of the contents and material related to each lesson as well as structured questions about the delivery of each lesson for integrity evaluation.

#### Content

The structure and contents of the HE lessons in the intervention group were guided by the Health Action Process Approach (HAPA)-model [23] and are introduced in Table 1.

**Table 1:** The structure and contents of the health education lessons guided by the Health Action Process approach [23]

Procedure	Contents	Elements of HAPA
Orientation Phase		
Lesson 1: Orientation	- Teacher presents the intervention and informs about the SoftGIS questionnaire	- attitudes
	- Students complete the SoftGIS questionnaire via internet (https://softgis.org.aalto.fi/childrens/questions/begin)	
	- Homework 1 for the next lesson: FeetEnergy-homework leaflet and instructions	
Motivational Phase: Intention building		
Homework 1 - Me & PA	- FeetEnergy-homework leaflet, part 1: Self-assessment of time spent in a) active commuting to school, b) moderate-intensity physical activity (PA) and c) sedentary behavior (SB) and self-conclusion about meeting the recommendations for health.	- attitudes
Lesson 2: Me, peers & PA	- Teacher shows three feedback views based on the school-specific SoftGIS responses (Fig. 2).Teacher-led discussions on the views	- attitudes, outcome expectancies, pre-action self-efficacy, intention
	View 1: active commuting to school	
	Map of the city of Tampere is shown with a dot indicating the school ⇒ proportion of students by gender and an average minutes of walking or cycling to school within 4 distance circles from home (less than 1 km, 1-3 km, 3-5 km, more than 5 km) is shown	
	View 2: leisure time PA	- action planning, action self-efficacy
	The quantity of moderate-intensity LTPA is shown by average and by sex	
	View 3: screen time	
	The proportion of students meeting the recommendation of screen-viewing up to 2 hours are shown by average and by sex	
	- Homework for the next lesson: Link to FeetEnergy -video (www.youtube.com/watch?v=Q22XOs1DEtM) and instructions	
Volitional Phase: Action Planning		
Homework 2 - Recognizing one’s possibilities	- Watching FeetEnergy-video, which introduces PA recommendations and gives tips for increasing PA and decreasing SB	- action planning, action self-efficacy
	- FeetEnergy-homework leaflet, part 2: Making a list of self-selected ways to increase PA and to reduce SB and choosing at least one way for an immediate action plan	
Lesson 3: Goal setting and action planning	- Watching the FeetEnergy –video in the classroom	- action self-efficacy
	- Discussing in small groups or pairs about the self-selected ways for immediate action plan.	
	- Making the actions visible by writing them on the FeetEnergy-classroom poster	
	- Homework for the next lesson: Writing follow-up comments about the realization of the actions to the space provided in the poster (brief summary in the beginning of lesson 4)	

#### 1st lesson

The first lesson was implemented in the computer class of the school under the supervision of the HE teacher. First the students completed the internet-based SoftGIS questionnaire individually at https://softgis.org.aalto.fi/childrens/questions/begin. The SoftGIS inquiry uses the Geographic Information System (GIS) to enable mapping of e.g. schools routes, PA places, modes of transportation and social interactions in specific places. Time spent in leisure PA and SB are also elicited with conventional questions. The SoftGIS has been tested in several Finnish cities and a series of usability studies have been conducted to develop it for children and young people [24, 25]. SoftGIS questionnaire was included in the intervention because it was an easily transformable internet-based tool particularly planned for the target population. In addition, not many studies using Internet in PA promotion have been reported internationally [20]. In Finnish schools Internet is still quite unutilized resource in teaching despite relatively high skills in information and communication technology among young people and good availability of wireless connections in schools. [26].

At the end of the lesson the students received a FeetEnergy-homework leaflet (Fig. 2). It included self-assessment of PA and SB and information on PA and SB from the health perspective as well as space for listing self-selected ways to increase PA and reduce SB and for choosing at least one way for an immediate action plan. The students were to complete the self-assessment for the next lesson as homework. They were also provided a similar leaflet to be delivered to their parents. The parental FeetEnergy leaflet guided the parents to assess the amount of their adolescent’s PA and SB, included information on PA and SB from the health point of view and introduced ways the parents could encourage their adolescents to increase PA and to reduce SB.

#### 2^nd^ lesson

**Fig. 3:**
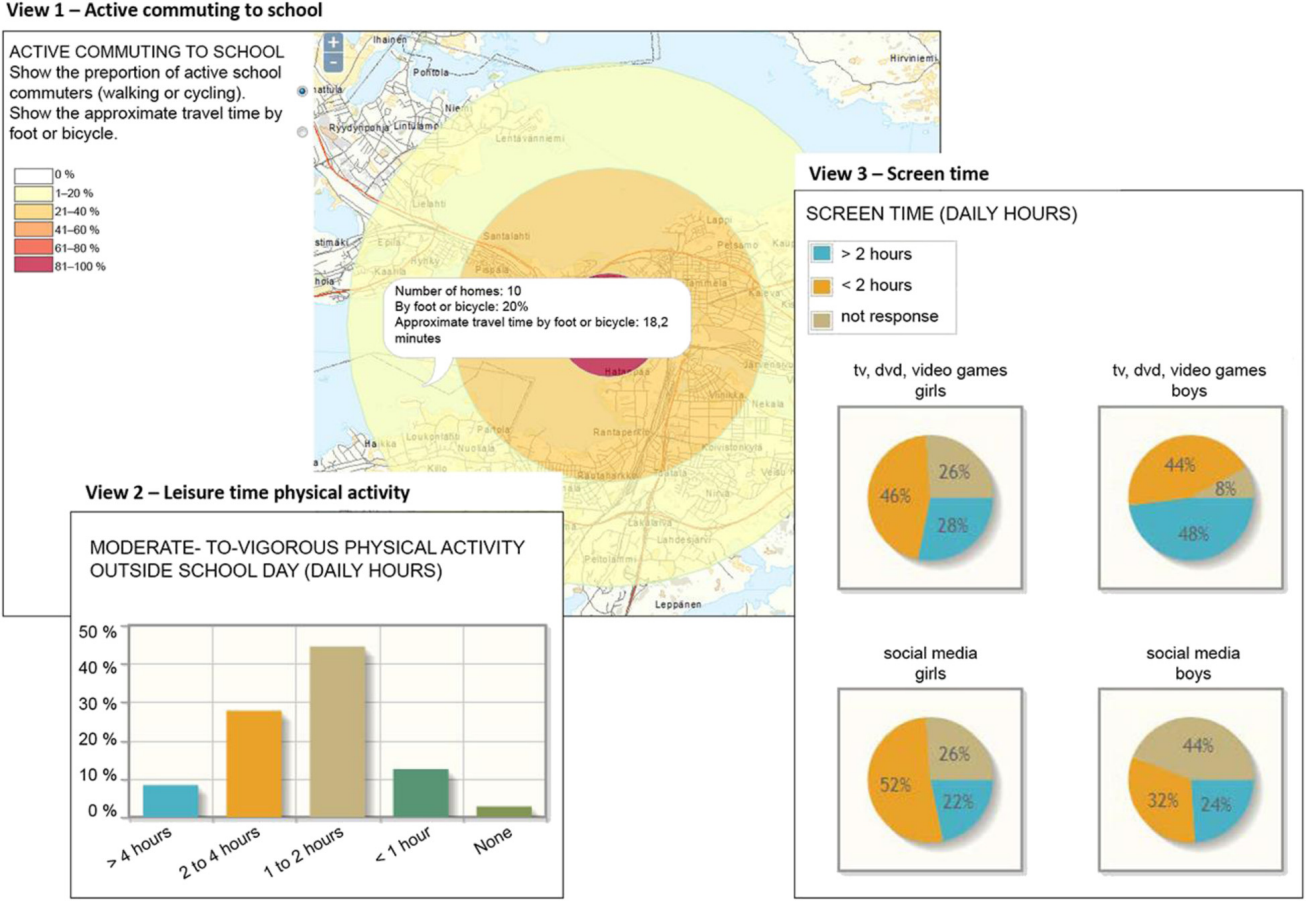
School-specific feedback views from SoftGIS responses

The second lesson started with discussions about the school-specific feedback views accumulated automatically from the responses to SoftGIS questionnaire (Fig. 3). The three views gave the students an overview on their active commuting to school in relation to three different distance categories from home (View 1), on the proportion of students falling into four different categories of weekly duration of moderate-intensity leisure PA (View 2) and on the proportion of students spending daily less than two hours and two hours or more on screen time related to TV, dvd, computer games and social media. (View 3).

At the end of the lesson the students received a link to a YouTube video www.youtube.com/watch?v=Q22XOs1DEtM offering information and ideas for being more physically active and reducing sitting in everyday life. After watching the video as homework the students were to complete rest of the FeetEnergy-leaflet received during the 1^st^ lesson (self-selected list of ways to increase PA and to reduce SB and choosing at least one way for an immediate action plan).

#### 3^rd^ lesson

At the beginning of the lesson the class watched the FeetEnergy-video together and discussed in small groups or pairs about the ways they had entered to their personal action plans. In the end of the discussions the teacher hung a FeetEnergy-poster (Fig. 2) on the classroom wall for the students to make their actions plans visible. The lesson ended with the teacher’s brief summary about the ways listed in the poster and to the encouragement for writing down follow-up comments about the realization of the action plans to the space provided in the poster. At the beginning of next HE lesson the teachers led a brief discussion about the comments.

#### Comparison group

In the comparison group the HE teachers were requested, if necessary, to adjust the HE lessons on PA to the same time period as in the intervention group. None of the teachers declined from this request. In the comparison group (n = 855), only data collection was carried out and the teachers received the intervention material after the intervention.

## Results

The evaluation of the intervention is based on the RE-AIM framework developed by Glasgow et al. [21] and including five dimensions: **R**each, **E**ffectiveness, **A**doption, **I**mplementation and **M**aintenance (Table 2). It is recommended for the evaluation of health promotion interventions for more systematic balancing of internal and external validity [27, 28], which is needed for translating the study results into practice. In Finland, the framework has been used e.g. in the evaluation of national physician-based PA promotion program [29].

**Table 2:** Evaluation questions and indicators of the intervention based on RE-AIM framework

Dimension	Evaluation question	Indicator
Reach	What percentage of potentially eligible participants will take part and how representative are they?	- number and representativeness of the adolescents participating
Effectiveness	What impact did the intervention have on psychosocial factors of physical activity (PA) and sedentary behavior (SB)?	- social norms, intentions, self-efficacy
	What impact did the intervention have on PA and SB?	- weekly frequency of active commuting to school
		- weekly frequency and minutes of leisure PA of various intensities
		- weekly minutes of daily sitting
		- meeting the daily recommendation of moderate-to-vigorous-intensity PA (1 hour on 7
		days per week)
		- meeting the daily screen time recommendation (≤ 2 hours on 7 days per week)
	What impact did the intervention have on parental interference with their child’s PA and SB?	- number of parents interfering with their child’s PA and SB
Adoption	What percentage of settings and intervention agents participated and how representative were they?	- number and representativeness of the schools participating
Implementation	To what extent were the various intervention actions delivered as intended?	- number of teachers delivering the intervention
		- number of health education lessons realized as intended
		- proportion of students exposed to the intervention during the health education lessons
		- number of parents recalling the parental FeetEnergy-leaflet
	To what extent did the participants have adverse effects related to active commuting to school, LTPA and sitting?	- number of students with PA restrictions
Maintenance	What were the long-term effects? (Individual level)	- same indicators as in effectiveness
	To what extent were the intervention actions maintained? (Setting level)	- use of intervention material by the health education teachers 12 months after the
		intervention

### Reach

Reach is assessed by dividing the number of students participating in the HE intervention lessons by the total number of 8^th^ graders in the participating schools. Representativeness is assessed by comparing the baseline information (age, gender, PA etc.) reported by the participants with information obtained from national surveys of this age group.

#### Effectiveness

Adolescents’ PA and SB are assessed with a questionnaire and an accelerometer (Hookie AM20, Traxmeet Ltd., Espoo, Finland, www.traxmeet.com) at baseline (one week before the intervention), 4 weeks after the intervention and 7 months after the baseline. The last follow-up point indicates long-term effectiveness (individual level maintenance). The questionnaires were delivered to the school secretaries approximately one week before measurements and collected two weeks later. The questionnaires were completed during any lesson under the supervision of a teacher. The questionnaire included questions about different modes of school transportation, leisure PA and SB.

Accelerometer was offered to all 8^th^ graders at the same measurement points as the questionnaire for more objective between-group comparison of PA and SB. The students (N = 404) were instructed to wear the accelerometer during waking hours for 7 days (from Monday to Sunday). Accelerometer data was linked to the daily contexts with the help of a 7-day activity diary including wake-up and going-to-sleep time, time going to and coming back from school as well as times starting and ending physical education lessons, structured sports activities and other leisure PA. The amount of screen time and other SB was also recorded daily. Brief verbal information about the measurement protocol was provided for the students by the teachers or research staff when delivering the questionnaire and accelerometer to them. SMS message was sent to the students agreeing to use accelerometer and/or to one of their parents each day at 9 p.m. as a reminder to use the accelerometer on the next day and completing the diary entries of the same day. Students’ mobile numbers were obtained from their informed consents.

Psychosocial factors (norms, intentions, self-efficacy) related to PA and SB were assessed as intervention outcomes in addition to students’ actual PA and SB behavior due to the shortness of the intervention. They are known to act as moderators and mediators of PA [30–32] and have been shown to affect adolescents’ PA behavior [33–36]. However, no studies in adolescents using these specific factors as intervention outcomes have been reported in Finland. Therefore, the questions included in the baseline and follow-up questionnaires were developed by utilizing earlier studies conducted elsewhere [37–42].

The frequency of parental interference with their child’s PA and SB was also used as outcome of effectiveness because parents’ valuation of PA and personal habits have been found to associate with their children’s PA [13]. Parents received a questionnaire via their children at baseline and four weeks after the intervention. The students returned the completed questionnaires in the sealed envelopes back to schools. The parental questionnaire included questions on the family background, parents’ PA and SB, parental perceptions about their children’s PA and SB and family discussions about PA and SB.

#### Adoption

The number of participating schools in relation to total number of schools recruited indicated adoption. It was assessed with recruitment notes kept by the researchers. Representativeness was assessed by comparing the participating schools with all the schools recruited.

#### Implementation

The realization of HE lessons was obtained from the records kept by the HE teachers in the Teacher’s Manual. The teachers were also interviewed after the intervention to get information about the acceptability of the material they used during the lessons. Parents were elicited about recalling the FeetEnergy-leaflet in the follow-up questionnaire at 4 weeks after the intervention.

#### Maintenance

The sustainability of the possible changes in adolescents’ PA and SB was assessed with the last follow-up questionnaire and accelerometer at 7 months from baseline (individual level). The HE teachers of the intervention group were e-mailed an electronic questionnaire two years after the beginning of the intervention about the extent they were using the material developed for the HE lessons (organizational level).

### Statistical methods

The main method in the comparison of the intervention and the comparison group is generalized linear mixed models (GLMM). In the analysis student-level, teacher-level and school-level influences on the outcomes can be examined simultaneously and the results are corrected for between-school and between-teacher variation. The choice of the link function in GLMM depends on the measurement scale and distribution of the outcome variable. Intention-to-treat principle cannot be followed because no information is available on those students not completing the questionnaires or not using the accelerometer. Drop-out rate can be determined by comparing the number of students in each school with the number of students completing the questionnaire and using the accelerometer.

## Discussion

This paper gives an overview of the protocol and evaluation of Kids Out! –study, which is the first in Finland to utilize HE lessons in PA promotion. It is hypothesized that the intervention has positive effects on 8^th^ graders’ PA and SB, psychosocial determinants of PA and SB and parents’ interference with their children’s PA and SB and that it can be feasibly implemented in routine school work. The transferability of the results into practice is strengthened by the RE-AIM evaluation, which targets not only effectiveness but also to the external validity of the intervention. Analyzing the results has started and several articles will be prepared for international peer-reviewed journals.

If the results of the intervention are encouraging, it may be an indication that integration of simple structured elements into routine school practices is worth considering also in larger scale. As a side effect, the study produces educational material for upper graders, which is needed in Finland, where most available material for PA promotion is for children. At best the study may give a broad perspective in developing evidence-based and practice-based multi-component intervention to promote PA and to reduce SB in school settings.

## Abbreviations

PA: physical activity
SB: sedentary behavior
HE: health education
GLMM: generalized linear mixed models

## References

[1] Strong WB, Malina RM, Blimkie CJR, Daniels SR, Dishman RK, Gutin B, Hergenroeder AC, Must A, Nixon PA, Pivarnik JM, Rowland T, Trost S, Trudeau F (2005). Evidence based physical activity for school-age youth. J Pediatr..

[2] Physical Activity Guidelines Advisory Committee. Physical Activity Guidelines Advisory Committee Report, 2008. Washington, DC: U.S. Department of Health and Human Services; 2008. www.health.gov/paguidelines/

[3] Janssen I, LeBlanc AG (2010). Systematic review of the health benefits of physical activity and fitness in school-aged children and youth. Int J Behav Nutr Phys Act..

[4] Landry BW, Driscoll SW (2012). Physical activity in children and adolescents. PM&R..

[5] Singh A, Uijtdewilligen L, Twisk J, van Mechelen W, Chinapaw M (2012). Physical activity and performance at school. A systematic review of the literature including a methodological quality assessment. Arch Pediatr Adolesc Med.

[6] Currie C, Zanotti C, Morgan A, Currie D, de Looze M, Roberts C, Samdal O, Smith ORF, Barnekow V (2012). 2012. Social determinants of health and well-being among young people. HBSC international report from the 2009/2010 survey. Health Policy for Children and Adolescents, No. 6.

[7] Tammelin T, Laine K, Turpeinen T (eds.). Oppilaiden fyysinen aktiivisuus. Jyväskylä: Liikunnan ja kansanterveyden julkaisuja 272;. Jyväskylä 2013. (In Finnish)

[8] Pate RR, Mitchell JA, Byun W, Dowda M (2011). Sedentary behaviour in youth. Br J Sports Med..

[9] Booth FW, Hargreaves M (2011). Understanding multi-organ pathology from insufficient exercise. J Appl Physiol..

[10] Tremblay M, LeBlanc A, Kho M, Saunders T, Larouche R, Colley R, Goldfield G, Connor Gorber S (2011). Systematic review of sedentary behavior and health indicators in school-aged children and youth. Int J Behav Nutr Phys Act..

[11] Fogelholm M, Paronen O, Miettinen M (2007). Physical activity – a possibility for welfare policy. The state and development of health-enhancing physical activity in Finland.

[12] Buliung RN, Mitra R, Faulkner G (2009). Active school transportation in the Greater Toronto Area, Canada: an exploration of trends in space and time (1986–2006). Prev Med..

[13] Davison KK, Werder JL, Lawson CT (2008). Children’s active commuting to school: current knowledge and future directions. Prev Chronic Dis..

[14] The National Travel Survey 2011-2012. Ministry of Transport and Communications Finland, the Finnish National Road Administration and the Finnish Rail Administration; 2012. http://portal.liikennevirasto.fi/portal/page/portal/e/fta/research_development/national_travel_survey/HLT_2010_2011_esite_ENG_0.pdf

[15] Cooper AR, Page AS, Wheeler BW, Griew P, Davis L, Hillsdon M, Jago R (2010). Mapping the walk to school using accelerometry combined with a global positioning system. Am J Prev Med..

[16] Faulkner GEJ, Buliung RN, Flora PK, Fusco C (2009). Active school transport, physical activity levels and body weight of children and youth: aA systematic review. Prev Med..

[17] Larouche R, Saunders TJ, Faulkner G, Colley R, Tremblay M (2014). Associations between active school transport and physical activity, bodycomposition, and cardiovascular fitness: a systematic review of 68 studies. J Phys Act Health.

[18] Dobbins M, DeCorby K, Robeson P, Husson H, Tirilis D (2013). School-based physical activity programs for promoting physical activity and fitness in children and adolescents aged 6-18. Cochrane Database Syst Rev..

[19] Kriemler S, Meyer U, Martin E, van Sluijs EM, Andersen LB, Martin BW (2011). Effect of school-based interventions on physical activity and fitness in children and adolescents: a review of reviews and systematic update. Br J Sports Med..

[20] Murillo Pardo B, García Bengoechea E, Generelo Lanaspa E, Bush PL, Zaragoza Casterad J, Julián Clemente JA, García GL (2013). Promising school-based strategies and intervention guidelines to increase physical activity of adolescents. Health Educ Res.

[21] Glasgow RE, Vogt TM, Boles SM (1999). Evaluating the public health impact of health promotion interventions: tThe RE-AIM framework. Am J Public Health..

[22] Paronen O, Aittasalo M, Jussila A. Kasit liikkeelle! Koulumatka ja liikuntakysely Tampereella 2011. http://www.tampere.fi/material/attachments/k/6BHKihkw0/Kasit_liikkeelle_Koulumatka-_ja_liikuntakysely_Tampereella.pdf. (In Finnish)

[23] Schwarzer R. Modeling health behavior change: how to predict and modify the adoption and maintenance of health behaviors. Appl Psychol. 2008;57:1–29.

[24] Kyttä M, Kahila M (2006). PehmoGIS elinympäristön koetun laadun kartoittajana (SoftGIS methodology in revealing the localized experiences of living environment).

[25] Kyttä M, Kahila M, Broberg A, Tynnilä J. Laatu kokemuksina (Quality as an experience). In Staffans, A, & Väyrynen, E,. (eds.) Oppiva kaupunkisuunnittelu (Urban Plannign as a Learning Process). Helsinki University of Technology, Department of Architecture. Publication 98;79-120;, 2009b. (In Finnish)

[26] European Schoolnet and University of Liege. (2013). Survey of schools: ICT in education. Benchmarking access, use and attitudes to technology in Europe’s schools. Final Report (ESSIE).Brussels: European Union. Retrieved November 26 2013. http://ec.europa.eu/digital-agenda/sites/digital-agenda/files/KK-31-13-401-EN-N.pdf.

[27] Eakin EG, Smith BJ, Bauman A (2005). Evaluating the population health impact of physical activity interventions in primary health care – are we asking the right questions?. J Phys Act Health..

[28] Owen N, Glanz K, Sallis JF, Kelder SH (2006). Evidence-based approaches to dissemination and diffusion of physical activity interventions. Am J Prev Med.

[29] Aittasalo M, Miilunpalo S, Ståhl T, Kukkonen-Harjula K (2007). From innovation to practice: initiation, implementation and evaluation of a national physician-based physical activity promotion programme. Health Promot Int..

[30] Ridley K, Olds TS, Hill A (2006). The Multimedia activity recall for children and adolescents (MARCA): development and evaluation. Int J Behav Nutr Phys Act..

[31] Bandura A (2004). Health promotion by social cognitive means. Health Educ Behav..

[32] Dishman RK, Mot RW, Saunders R, Felton G, Ward DS, Dowda M (2004). Self-efficacy partially mediates the effect of a school-based physical-activity intervention among adolescent girls. Prev Med..

[33] Dishman RK, Saunders RP, Motl RW, Dowda M, Pate RR (2009). Self-efficacy moderates the relation between declines in physical activity and perceived social support in high school girls. J Pediatr Psych..

[34] De Bourdeaudhuij I, Lefevre J, Deforche B, Wijndaele K, Matton L, Philippaerts R (2005). Physical activity and psychosocial correlates in normal weight and overweight 11 to 19 year olds. Obes Res..

[35] Haerens L, Cerin E, Maes L, Cardon G, Deforche B, De Bourdeaudhuij I (2008). Explaining the effect of a 1-year intervention promoting physical activity in middle schools: a mediation analysis. Public Health Nutr.

[36] Spence JC, Blanchard CM, Clark M, Plotnikoff RC, Storey KE, McCargar LJ (2010). The role of self-efficacy in explaining gender differences in physical activity among adolescents: A multilevel analysis. J Phys Act Health..

[37] Van Der Horst K, Paw MJCA, Twisk JWR, Van Mechelen W (2007). A brief review on correlates of physical activity and sedentariness in youth. Med Sci Sports Exerc.

[38] Deforche B, De Bourdeaudhuij I, Tanghe A, Hills AP, De Bode P (2004). Changes in physical activity and psychosocial determinants of physical activity in children and adolescents treated for obesity. Patient Educ Couns..

[39] Dishman RK, Hales DP, Sallis JF, Saunders R, Dunn AL, Bedimo-Rung AL, Ring KB (2010). Validity of social-cognitive measures for physical activity in middle-school girls. J Pediatr Psych..

[40] Jago R, Baranowski T, Watson K, Bachman C, Baranowski JC, Thompson D, Hernández AE, Venditti E, Blackshear T, Moe E (2009). Development of new physical activity and sedentary behavior change self-efficacy questionnaires using item response modeling. Int J Behav Nutr Phys Act..

[41] Lee RE, Nigg CR, DiClemente CC, Courneya KS (2001). Validating motivational readiness for exercise behaviour with adolescents. Res Q Exerc Sport..

[42] Motl RW, Dishman RK, Trost SG, Saunders RP, Dowda M, Felton G, Ward DS, Pate RR (2000). Factorial validity and invariance of questionnaires measuring social-cognitive determinants of physical activity among adolescent girls. Prev Med..

